# The effect of different dialysate sodium concentrations on ambulatory blood pressure in hemodialysis patients: a prospective interventional study

**DOI:** 10.1093/ckj/sfae041

**Published:** 2024-02-21

**Authors:** Adamantia Bratsiakou, Fotini Iatridi, Marieta Theodorakopoulou, Pantelis Sarafidis, Dimitrios S Goumenos, Evangelos Papachristou, Marios Papasotiriou

**Affiliations:** Department of Nephrology and Kidney Transplantation, University Hospital of Patras, Patras, Greece; First Department of Nephrology, Hippokration Hospital, Aristotle University of Thessaloniki, Thessaloniki, Greece; First Department of Nephrology, Hippokration Hospital, Aristotle University of Thessaloniki, Thessaloniki, Greece; First Department of Nephrology, Hippokration Hospital, Aristotle University of Thessaloniki, Thessaloniki, Greece; Department of Nephrology and Kidney Transplantation, University Hospital of Patras, Patras, Greece; Department of Nephrology and Kidney Transplantation, University Hospital of Patras, Patras, Greece; Department of Nephrology and Kidney Transplantation, University Hospital of Patras, Patras, Greece

**Keywords:** ABPM, blood pressure, dialysate sodium concentration, three-day interdialytic interval

## Abstract

**Background:**

Hypertension is associated with increased morbidity and mortality in hemodialysis patients. Existing recommendations suggest reduction of sodium load, but the effect of dialysate sodium on blood pressure (BP) is not fully elucidated. The aim of the present study is to investigate the effect of different dialysate sodium concentrations on 72-h ambulatory BP in hemodialysis patients.

**Methods:**

This prospective study included patients on standard thrice-weekly hemodialysis. All patients initially underwent six sessions with dialysate sodium concentration of 137 meq/L, followed consecutively by another six sessions with dialysate sodium of 139 meq/L and, finally, six sessions with dialysate sodium of 141 meq/L. At the start of the sixth hemodialysis session on each sodium concentration, 72-h ABPM was performed over the long interdialytic interval to evaluate ambulatory systolic and diastolic BP (SBP and DBP) during the overall 72-h, different 24-h, daytime and night-time periods.

**Results:**

Twenty-five patients were included in the final analysis. A significant increase in the mean 72-h SBP was observed with higher dialysate sodium concentrations (124.8 ± 16.6 mmHg with 137 meq/L vs 126.3 ± 17.5 mmHg with 139 meq/L vs 132.3 ± 19.31 mmHg with 141 meq/L, *P *= 0.002). Similar differences were noted for DBP; 72-h DBP was significantly higher with increasing dialysate sodium concentrations (75.1 ± 11.3 mmHg with 137 meq/L vs 76.3 ± 13.7 mmHg with 139 meq/L vs 79.5 ± 13.9 mmHg with 141 meq/L dialysate sodium, *P *= 0.01). Ambulatory BP during the different 24-h intervals, daytime and night-time periods was also progressively increasing with increasing dialysate sodium concentration.

**Conclusion:**

This pilot study showed a progressive increase in ambulatory BP with higher dialysate sodium concentrations. These findings support that lower dialysate sodium concentration may help towards better BP control in hemodialysis patients.

KEY LEARNING POINTS **What was known:**

- Lower dialysate sodium concentration is associated with better BP in hemodialysis.
- Minimization of inter- and intradialytic sodium gain can lead to optimal BP control in hemodialysis patients.

**This study adds:**

- Lower dialysate sodium concentrations are associated with significant decrease in ambulatory 72-h BP.
- Lower dialysate sodium concentrations are associated with decreased intradialytic weight gain.

**Potential impact:**

- The use of lower dialysate sodium concentration may help towards better ambulatory BP control in hemodialysis patients.

## INTRODUCTION

Patients with end-stage kidney disease (ESKD) have increased risk for cardiovascular disease and mortality. Hypertension is the most common modifiable risk factor in this population, with an estimated prevalence around 85% [1]. One of the main contributors of elevated BP levels in patients with ESKD on hemodialysis is sodium and volume overload. Historically, the first hemodialysis treatments were performed with low dialysate sodium concentrations to achieve adequate sodium removal and BP control [2]. For several years, higher dialysate sodium concentrations (i.e. around 140 meq/L) were used to ensure better hemodynamic stability during the hemodialysis session, as intradialytic hypotension is also associated with higher mortality rates [3]. However, in observational studies higher dialysate sodium concentration is associated with increased thirst, higher BP levels and higher interdialytic weight gain (IDWG) [4–6]. Previous longitudinal studies have demonstrated that an individualized lower sodium concentration i.e. dialysate sodium concentration adjusted to the patient's plasma sodium (sodium set point), is associated with better BP control and lower IDWG [7, 8].

Recent studies suggest that hypertension management in hemodialysis patients is largely suboptimal, as only a minority is adequately controlled [9]. Existing recommendations focus first on non-pharmacological measures and thereafter on the use of proper medications to achieve proper BP control [1]. Among the former, achievement of correct dry weight is considered of utmost importance and randomized trials have recently showed that this measure can significantly affect ambulatory BP levels [10–12]. Another important recommendation related to minimization of inter- and intradialytic sodium gain includes decreasing dialysate sodium towards pre-dialysis sodium in selected individuals [1]. However, such suggestions have not been properly tested in clinical studies that included interdialytic BP measurements in hemodialysis patients.

Ambulatory BP monitoring (ABPM) is currently considered the gold standard for hypertension diagnosis and management in patients with ESKD [1]. Hemodialysis patients display different patterns of BP during the interdialytic interval [13]. The most commonly presented pattern is a rapid BP decrease during hemodialysis as a response to ultrafiltration, followed by a gradual rise during the interdialytic interval directly associated with IDWG [14]. The low accuracy and high variability of peridialytic BP measurements and the increasing sodium and water load during the interdialytic interval render ABPM the most reliable diagnostic method. Previous observations of significantly higher BP levels during the long day interval suggest that 48-h and 72-h ambulatory BP measurements provide valuable information regarding the trajectories and variability of BP levels that may be particularly helpful for hypertension management in these individuals [15–18].

As of this writing, only three studies assessed the effects of different dialysate sodium concentrations on ambulatory BP in the hemodialysis population [19–21], but all of them carried several limitations that gave birth to conflicting results and precluded generation of clear conclusions, while none of them used 72-h ABPM measurements. Given the scarcity of the relevant evidence in the field, we designed this study aiming to examine the effects of three different dialysate sodium concentrations on 72-h ambulatory BP levels during a whole 3-day interdialytic interval.

## MATERIALS AND METHODS

### Study population

This was a prospective, non-randomized interventional study conducted in the Hemodialysis Unit of the University Hospital of Patras, Greece, between September 2019 and December 2022. Inclusion criteria were: i) age > 18 years, ii) ESKD treated with a standard thrice-weekly hemodialysis schedule for longer than 3 months, and iii) informed written consent. Exclusion criteria were: (i) chronic atrial fibrillation or other arrhythmia that can interfere with ABPM; (ii) modification of antihypertensive medication during the month before study initiation; (iii) hemodynamic instability during the hemodialysis session, requiring intravenous fluids to restore BP, in >30% of sessions during the past three months; (iv) non-functional arteriovenous fistula at the contralateral brachial arm area of the one currently used for vascular access; (v) history of alcohol or drug abuse, or known severe mental disorder; and (vi) malignancy or any clinical condition associated with poor prognosis. All protocol procedures were approved by the Ethics Committee of School of Medicine, University Hospital of Patras (3607/24489) and were conducted in accordance with the Declaration of Helsinki (2013 Amendment).

### Study procedures and data collection

Participants that fulfilled the inclusion/exclusion criteria and consented to participate had a baseline evaluation of hydration status at the end of a mid-week hemodialysis session including physical examination and lung ultrasound. Blood samples were obtained for routine laboratory testing before the mid-week dialysis session. Demographics, anthropometric characteristics, comorbidities, concomitant medications, and dialysis-related parameters were recorded for every participant. All participants underwent a standard set of three periods of different dialysate sodium concentrations (each starting from the first session of the week), including initially six sessions with dialysate sodium concentration of 137 meq/L, followed by six sessions with dialysate sodium concentration of 139 meq/L and finally six sessions with dialysate sodium concentration of 141 meq/L. Thus, according to study design every patient should complete the study procedures in six consecutive weeks (i.e. 42 days). All patients were dialyzed with standard synthetic membranes, with blood flow rate ranging between 300–400 ml/min and dialysate flow rate of 500 ml/min; ultrafiltration volume was calculated according to their pre-defined dry weight, based on standard clinical criteria. Participants were instructed to follow their usual activities, including physical activity, sleep, food and water intake, and follow their medication with no deviations over the study period. Dry weight and antihypertensive medication were not allowed to change during the study.

### Assessments

Ambulatory BP was evaluated with the Mobil-O-graph NG device (IEM, Stolberg, Germany), an oscillometric ABPM device that has been validated for brachial BP measurement according to standard protocols [22], and that provides the same values as a widely used ABPM monitor [23]. The device was placed on the non-fistula arm using appropriate-size cuffs. The recording began at the start of the sixth session at each different dialysate sodium period, which was the last session of the week, and was programmed to cover a whole 72-h intra- and interdialytic interval. The monitor was set to measure BP every 20 minutes during daytime (07:00 to 22:59) and every 30 minutes during night-time (23:00 to 06:59). Recordings were included in the analysis only if >80% of the readings were valid, with a maximum of two non-consecutive day-time hours with fewer than two valid measurements, and a maximum of one night-time hours with no valid measurement. If a valid recording was not performed at first instance patients continued on the same dialysate sodium concentration for another week and the ABPM was repeated (in this case the total study duration was increased to 7 consecutive weeks (i.e. 49 days)). If the ABPM was still not valid, the patients were excluded from further analysis. In order to minimize the possible effect of manual BP measurements, only measurements recorded at the prespecified time intervals at which the device was set to measure BP were used in this analysis and all manual measurements were excluded.

Lung ultrasound was performed at the end of a mid-week hemodialysis session, using the SonoScape Co Model S2 SONOMED device. Participants were assessed with the classical Jambrik 28-sites score and the total number of US B-lines were recorded [24]. Body weight was actively monitored before and after each hemodialysis session, to identify the effect of different dialysate sodium concentrations on IDWG during the long interdialytic interval.

### Statistical analysis

All statistical analyses were performed with GraphPad Prism 8 software. Continuous variables are presented as mean and standard deviation (SD) or median and interquartile range [IQR] depending on the normality of distribution, which was assessed with the Shapiro–Wilk test and confirmed by visual assessment of curves in histograms. Categorical data are presented as frequencies and percentages (*n*, %). To investigate the effect of different dialysate sodium concentrations in ambulatory BP between the three periods, one-way analysis of variance ANOVA for repeated measurements was performed. To examine the effect of sodium dialysate concentration and test for possible differences between anuric and non-anuric patients (defined as those with urine output ≥500 ml/24 h), as well as determine whether an interaction between these two variables exist, we used two-way mixed ANOVA for repeated measurements. A *P*-value level <0.05 was considered statistically significant.

## RESULTS

The flowchart of study participants is provided in Fig. S1 (see online supplementary material). From a total of 70 assessed, 39 patients were found eligible according to the inclusion/exclusion criteria and provided written consent to participate. Twenty participants had to repeat one measurement due to invalid first recording; nine of them successfully completed the measurements, while 11 had invalid measurements for a second time and had to be excluded. One patient suffered a myocardial infarction and was admitted to the ICU and two patients denied completing the study procedures. Finally, a total of 25 patients (21 men and four women) were included in the analysis. Demographic and anthropometric characteristics, comorbidities, laboratory tests and antihypertensive medication of study participants are presented in Table 1. IDWG increased with higher dialysate sodium concentrations (2.62 ± 0.92 kg with 137 meq/L vs 2.67 ± 1.3 kg with 139 meq/L vs 3.06 ± 1.17 kg with 141 meq/L, *P *= 0.076), although this difference did not reach statistical significance.

**Table 1:** Demographic and anthropometric characteristics, comorbidities and antihypertensive medication of study participants.

**Ν**	25
Age (years)	59.4 ± 12.9
Male gender (*n*, %)	21(84%)
Dry body weight (Kg)	78.4 ± 1.2
Height (cm)	170.9 ± 8.1
ΒΜΙ (Κg/m²)	26.81 ± 4.49
Hemodialysis vintage (months)	38.4 ± 36.4
Urine output >500 ml/24 h (*n*, %)	9 (36%)
**Comorbidities**	
Diabetes (*n*, %)	13 (52%)
Hypertension (*n*, %)	22 (88%)
Dyslipidemia (*n*, %)	17 (68%)
Coronary heart disease (*n*, %)	7 (28%)
Ηeart failure (*n*, %)	8 (32%)
Stroke (*n*, %)	1 (4%)
Peripheral vascular disease (*n*,%)	9 (36%)
**Antihypertensive medication**	
Use of antihypertensive drugs (*n*, %)	20 (80%)
1 antihypertensive drug (*n*, %)	6 (24%)
2 antihypertensive drugs (*n*, %)	11 (44%)
3 antihypertensive drugs (*n*, %)	3 (12%)
ACE inhibitors (*n*, %)	1 (4%)
ARBs (*n*, %)	5 (20%)
β-blockers (*n*, %)	17 (68%)
CCBs (*n*, %)	10 (40%)
Centrally acting agents (*n*, %)	2 (8%)
Nitrates (*n*, %)	3 (12%)
Loop diuretics (*n*, %)	6 (24%)
US B-lines (number)	11 ± 4.1
**Laboratory values**	
Serum sodium (mmol/L)	137.0 ± 3.0
Serum potassium (mmol/L)	5.1 ± 0.6
Serum urea (mg/dL)	160.7 ± 33.2
Serum creatinine (mg/dL)	8.1 ± 1.9
Hemoglobin (g/dL)	10.5 ± 0.9

Continuous data are presented as mean and standard deviation (SD) or median and interquartile range [IQR]. Categorical data are presented as frequencies and percentages (n,%).

BMI, body mass index; UF, ultrafiltration; IDWG, interdialytic weight gain; ACE, angiotensin converting enzyme; ARBs, angiotensin receptor blockers; CCBs, calcium channel blockers; US, ultrasound.

### Comparison of ambulatory BP levels according to different dialysate sodium concentrations

**Figure 1:**
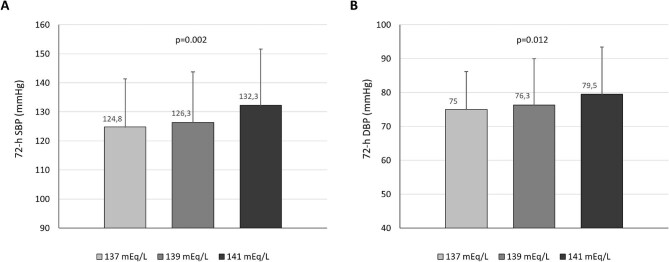
72-h SBP and DBP at each dialysate sodium concentration Data are presented as mean and standard deviation (SD).

Ambulatory SBP and DBP parameters during the 3-day interdialytic interval, according to the three different dialysate sodium concentrations are shown in Table 2. The levels of 72-h SBP at each dialysate sodium are depicted in Fig. 1A. Overall, 72-hour SBP was significantly higher with increasing dialysate sodium concentrations (124.8 ± 16.6 mmHg with 137 meq/L vs 126.3 ± 17.5 mmHg with 139 meq/L vs 132.3 ± 19.31 mmHg with 141 meq/L, *P *= 0.002). Similarly, 72-h DBP was significantly higher with higher dialysate sodium concentrations (75.1 ± 11.3 mmHg with 137 meq/L vs 76.3 ± 13.7 mmHg with 139 meq/L vs 79.5 ± 13.9 mmHg with 141 meq/L dialysate sodium, *P *= 0.01) (Table 2 and Fig. 1B). The mean values of 72-h SBP and DBP and the pattern of change for each patient over the three different dialysate sodium concentrations are presented in Fig. S2 (see online supplementary material).

**Table 2:** Ambulatory BP levels during the whole 72-h and the respective 24-h periods, with the three different dialysate sodium concentrations.

**Parameter**	**Na 137 meq/L**	**Na 139 meq/L**	**Na 141 meq/L**	***P***
72-h SBP (mmHg)	124.8 ± 16.6	126.3 ± 17.5	132.3 ± 19.3	0.002
72-h DBP (mmHg)	75.0 ± 11.2	76.3 ± 13.7	79.5 ± 13.9	0.012
1st 24-h SBP (mmHg)	121.1 ± 18.2	122.8 ± 20	128.1 ± 22.7	0.011
1st 24-h DBP (mmHg)	73.1 ± 11.9	75.1 ± 15.1	77.3 ± 15.6	0.049
2nd 24-h SBP (mmHg)	123.3 ± 16.4	125.3 ± 17.7	132.3 ± 19.3	<0.001
2nd 24-h DBP (mmHg)	73.8 ± 11.6	75.3 ± 13.6	79.5 ± 14.1	0.002
3rd 24-h SBP (mmHg)	130.2 ± 16.7	131.7 ± 16.6	137.5 ± 17.7	0.005
3rd 24-h DBP (mmHg)	78.3 ± 11	79 ± 13.2	81.9 ± 12.1	0.106

Data are presented as mean and standard deviation (SD)

SBP, systolic blood pressure; DBP, diastolic blood pressure

The relevant SBP and DBP levels during the different 24-h periods of the long interdialytic interval (first, second, and third) are also depicted in Table 2. Accordingly, in all studied periods, 24-h SBP was progressively increased with increasing dialysate sodium concentration (*P *< 0.05 for all comparisons). A similar trend was observed with DBP with significant changes observed for the first and second 24-period.

### Comparison of daytime and night-time BP levels according to different dialysate sodium concentrations

Blood pressure levels during daytime and night-time periods of the different time intervals are depicted in Table 3. Accordingly, ambulatory SBP levels during the various daytime and night-time periods of each interdialytic day were significantly higher with increasing concentrations of dialysate sodium, with the exception of intradialytic SBP.

**Table 3:** Ambulatory BP levels during the day-time and night-time periods of the three interdialytic days, with the three different dialysate sodium concentrations.

**Parameter**	**Na 137 meq/L**	**Na 139 meq/L**	**Na 141 meq/L**	***P***
Intradialytic SBP (mmHg)	130.2 ± 18	128.8 ± 21.2	135.3 ± 21.1	0.106
Intradialytic DBP (mmHg)	81.8 ± 12.8	81.7 ± 16.0	85.2 ± 14.9	0.27
1st day-time with HD DBP (mmHg)	74.6 ± 12	76.7 ± 15.1	78.8 ± 15.2	0.038
1st day-time without HD SBP (mmHg)	120.4 ± 19.8	122.6 ± 21.5	128.5 ± 24.2	0.009
1st day-time without HD DBP (mmHg)	72.3 ± 12.7	74.9 ± 15.8	77.1 ± 15.9	0.014
1st night-time SBP (mmHg)	117.5 ± 18.4	118.3 ± 20.6	124.1 ± 25.4	0.034
1st night-time DBP (mmHg)	69.3 ± 12.7	71.1 ± 16.0	73.7 ± 17.8	0.171
2nd day-time SBP (mmHg)	124.2 ± 16.6	126.5 ± 17.9	132.6 ± 19	0.005
2nd day-time DBP (mmHg)	74.2 ± 11.5	76.3 ± 13.9	80 ± 14.5	0.003
2nd night-time SBP (mmHg)	121.1 ± 16.9	121.8 ± 18.3	131.3 ± 21.9	0.001
2nd night-time DBP (mmHg)	72.8 ± 13	71.3 ± 12.6	78.4 ± 14.7	0.005
3rd day-time SBP (mmHg)	131.9 ± 17.6	132.5 ± 17.3	139.4 ± 18.5	0.007
3rd day-time DBP (mmHg)	79.2 ± 11.2	79.5 ± 13.8	83.2 ± 12.4	0.053
3^r^d night-time SBP (mmHg)	126.3 ± 15.9	129.9 ± 17.5	133.4 ± 18.5	0.009
3rd night-time DBP (mmHg)	76 ± 11.6	77.6 ± 13.2	79.2 ± 12.5	0.280

Data are presented as mean and standard deviation (SD)

HD, hemodialysis; SBP, systolic blood pressure; DBP, diastolic blood pressure

With regard to DBP, the observed increase during the whole 72-h period was, again, consistent across the various daytime and night-time periods, although differences in intradialytic DBP and DBP levels during the third day did not reach statistical significance.

### Comparison of differences in ambulatory BP levels between interdialytic days of the long interdialytic interval with the different dialysate sodium concentrations

**Figure 2:**
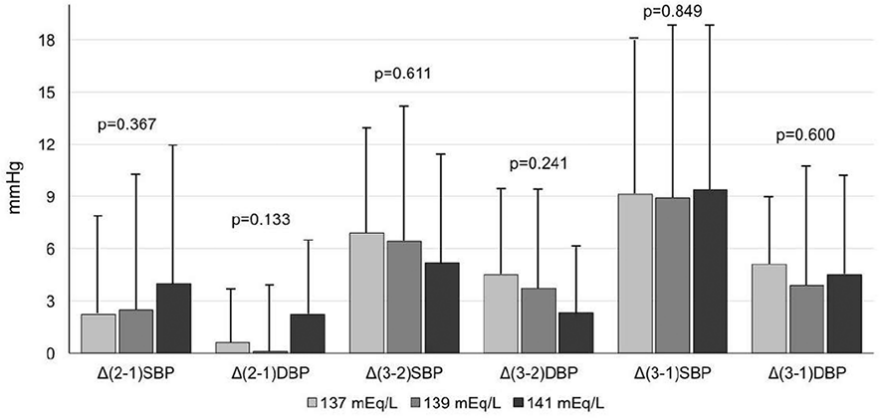
Differences (Delta, Δ) between the 1^st^ and 2^nd^, 2^nd^ and 3^rd^ and 1^st^ and 3^rd^ interdialytic days with each dialysate sodium Data are presented as mean and standard deviation (SD).

In order to examine whether the actual BP changes over different dialysate sodium levels were homogeneously distributed throughout the different 24-h periods of the long interdialytic interval, we have also calculated the differences (Delta, Δ) between the first and second, second and third, and first and third interdialytic days with each dialysate sodium. The BP changes between the 24-h, daytime and night-time periods of the interdialytic days with the three dialysate sodium concentrations are shown in Table S1 (see online supplementary material); changes between the respective 24-h periods with the different dialysate sodium concentrations are depicted in Fig. 2. Accordingly, 24-h SBP/DBP increased during the second compared to the first day and this change was even greater between second and third day; however, a different pattern of change was observed between the different sodium concentrations examined. The highest dialysate sodium was associated with higher BP increase from the first to the second interdialytic day compared with the lower dialysate sodium concentrations, while the highest BP increase from the second to the third day was observed with the lowest dialysate sodium concentration. The BP changes observed during the daytime and night-time periods between different interdialytic days displayed similar patterns across the different concentrations of dialysate sodium.

### Subgroup analysis according to urine output

**Figure 3:**
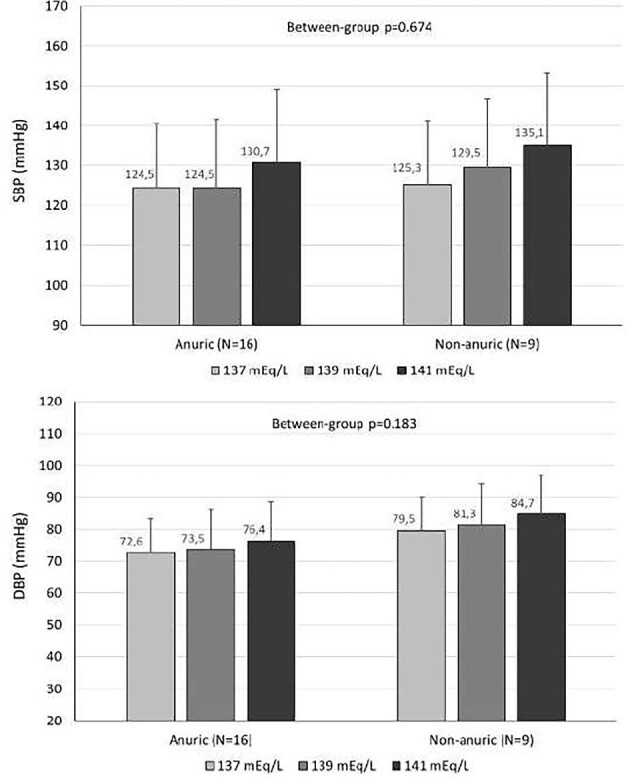
72-h SBP and DBP at each dialysate sodium concentration in groups stratified by urine output ≥500 ml/24 h. Data are presented as mean and standard deviation (SD).

With regard to 72-h SBP, there was a significant effect of sodium concentration (F[1, 22] = 16.843, *P *< 0.001, partial η^2 ^= 0.434), but not of urine output group, (F[1,22]=0.182, *P *= 0.674, partial η^2 ^= 0.008) or interaction between sodium concentration and group, F(1, 22) = 0.740, *P *= 0.399, partial η^2 ^= 0.033. For 72-h DBP, a significant effect of sodium concentration (F[1, 22] = 8.374, *P *= 0.008, partial η^2 ^= 0.276) was also evident, but not of urine output group, (F[1,22] = 1.889, *P *= 0.183, partial η^2 ^= 0.079) or interaction between sodium concentration and group, F(1, 22) = 0.537, *P *= 0.471, partial η^2 ^= 0.024 (Fig. 3).

## DISCUSSION

In this study we performed 72-h ABPM in a group of patients on maintenance HD, after the prescription of three different dialysate sodium concentrations to investigate potential differences in ambulatory BP between low and high dialysate sodium concentrations during the long interdialytic interval. The main findings of our study include significantly higher 72-h SBP/DBP levels with higher dialysate sodium concentration, with an observed difference of 7.5/4.46 mmHg between the highest and the lowest dialysate concentration. This finding of increased BP with higher sodium dialysate concentrations was consistent for all 24-h, daytime and night-time periods. We also examined the pattern of BP changes from one day of the long interdialytic interval to another. Although BP increase was numerically higher with all dialysate sodium concentrations from the second to the third compared to the first to the second 24-period, there was a different pattern of changes with the low dialysate sodium period showing the highest increase among groups from the second to the third day, whereas the high dialysate sodium period displayed the highest BP increase from the first to the second day. A notable increase in IDWG was observed with the highest dialysate sodium concentration of 141 meq/L.

Current knowledge suggests that higher dialysate sodium concentration improves hemodynamic stability during hemodialysis, minimizing the risk of hypotensive episodes; however, evidence suggest that increased sodium load can cause both intra and interdialytic hypertension [25, 26]. Sodium and volume overload represent a key contributing mechanism of hypertension, while several studies have investigated the effect of sodium on endothelial derived vasoregulators [27]. Furthermore, the intermittent nature of thrice-weekly hemodialysis leads to significant fluctuations in volume status, increasing the risk of cardiovascular complications [28]. In addition, earlier studies have demonstrated that the long interval is associated with elevated risk of cardiovascular death with the highest rate of death or hospitalization for cardiovascular causes observed the day after the 3-day interval. This higher mortality rate is mainly attributed to metabolic parameters like hyperkalemia and sodium and volume overload, especially in hemodialysis patients without residual kidney function [29].

Evidence deriving from recent studies suggests that although hypertension prevalence in hemodialysis patients reaches up to 85%, less than 30% has adequate BP control [9]. Based on current recommendations, non-pharmacological measures represent the first line treatment of hypertension in hemodialysis patients, followed by appropriate antihypertensive medications [1, 30]. Achievement of patients’ dry weight is currently considered a cornerstone of hypertension management, as dry weight probing has been associated with significantly lower ambulatory BP levels in recent randomized trials [10, 12]. Other important non-pharmacological measures recommended are related to minimization of inter- and intradialytic sodium gain, with suggestions of restriction of sodium intake to <65 mmol (1.5 g of sodium or 4 g of sodium chloride) per day, decreasing dialysate sodium towards pre-dialysis sodium in selected individuals and avoidance of sodium-containing or sodium-exchanging drugs [1]. However, these suggestions have not been properly tested in clinical studies with ABPM measurements, as happened in the case of dry weight probing.

During the past decades, several studies examined the effect of dialysate sodium concentration on BP, focusing mostly on intradialytic BP [20, 31–34]. In a randomized trial in 46 hemodialysis patients, the authors investigated the effect of low dialysate sodium on ambulatory BP. Patients were randomly assigned to receive hemodialysis treatment with low (137 meq/L) or high (140 meq/L) dialysate sodium for 6 months and 24-h ABPM was performed during the day after a mid-week session at two time-points, baseline and six months. Twenty-four-hour SBP, daytime and night-time SBP were significantly lower in low dialysate sodium group. One of the main advantages of this study was the long intervention period of six months. On the other hand, 24-h recordings do not provide adequate information about the BP changes during interdialytic intervals, as recording during intradialytic and first 20-h periods are absent; recordings during a whole intra- and inter-dialytic interval provide a better estimation of BP fluctuation patterns in hemodialysis patients [20]. In another study, Liu *et al.* randomized 64 hypertensive hemodialysis patients to 136 versus 138 mmol/l dialysate sodium concentration and measured 44-h ambulatory BP at baseline and 12 months later [21]. Although no difference in BP levels between the two groups was reported, there was a significant increase in antihypertensive medication in the control group. The observed difference in the intervention group was achieved without change in antihypertensive drugs, which indirectly supports a BP-lowering effect of low dialysate sodium. Both studies had conflicting findings and no firm conclusions could be drawn, as the certainty of the evidence was reported as low [20, 21, 35]. Furthermore, a recent randomized trial in 99 participants showed that low-sodium dialysate (135 meq/L) was not associated with significant improvements in BP levels after 12 months compared with conventional sodium dialysate (140 meq/L). However, this is a hypothesis-generating study (the primary outcome was LV mass index change) with several methodological issues. It included patients with ESKD on home or self-care satellite facility hemodialysis, a subset of patients that is far from being properly representative of the entire dialysis population as it exhibits a better BP control profile. Moreover, it used either ambulatory BP (unclear if this was 24-h or 44-h) or home BP measurements, but there are no data describing how many patients were ultimately assessed with each method; in addition, except for MAP, they did not report any results on SBP/DBP levels [19]. In another randomized crossover trial in 16 patients with intradialytic hypertension, Inrig *et al.* examined the effect of low dialysate sodium (5 meq/L lower than plasma sodium) versus high (5 meq/L higher than plasma sodium) on intradialytic BP. Low dialysate sodium was associated with lower one-week-average intradialytic SBP compared with high dialysate sodium concentration. However, only intradialytic measurements were included [36]. Finally, in one of the first studies that aimed to improve BP control in hemodialysis patients using lower dialysate sodium (gradual lowering from 140 mmol/L to 135 mmol/L at a rate of 1 mmol/L every 3–4 weeks) combined with dietary salt restriction (<6 g sodium chloride/day), pre-dialysis SBP/DBP showed a clear trend to reduce with the lower sodium concentrations without changes in dry weight [31]. All of these studies demonstrated that reduction in dialysate sodium concentration may significantly improve BP and arterial stiffness parameters. Our study expands the above observations, as it is not including exclusively peri- and intra-dialytic BP measurements and is the first among the very few studies using ABPM that investigated the effect of different dialysate sodium concentrations on the long interdialytic interval.

Studies regarding BP fluctuations over the long interdialytic interval are extremely scarce. A previous study evaluating 72-h BP and arterial stiffness parameters during the hemodialysis session and the long interval in 55 hemodialysis patients [15] showed that ambulatory aortic BP, Aix, and PWV levels were higher during the third day of the long interval compared with the second interdialytic day. These findings suggest a new pathway for increased cardiovascular risk during the third interdialytic day. Furthermore, dialysate sodium concentration has been shown to affect the total body water and sodium, so it can be hypothesized that higher dialysate sodium would be related to higher BP during the long interdialytic intervals. The findings of our study confirm this hypothesis and further add interesting data on the relevant increases of BP with different dialysate sodium concentrations over the different interdialytic days. The fact that the high dialysate sodium period displayed the highest BP increase from the first to the second day could be attributed to higher whole-body sodium and increased thirst in the first few hours after hemodialysis, leading to higher volume accumulation. This hypothesis-generating study needs to be further investigated with studies evaluating body weight, body composition, and serum osmolality over each day of the interdialytic interval.

Our study has strengths and limitations. It is the first interventional study comparing the effects of three different dialysate sodium concentrations on BP and the first study in the field using 72-h ABPM to assess BP changes throughout the long interdialytic interval. Ambulatory BP monitoring is always a difficult test, with feasibility that is reduced in haemodialysis patients [1, 37]; thus, studies with 72-hour recordings in this population are particularly scarce. A limitation of this pilot study is that we did not use a double-blind randomized design; this is difficult to do with sodium dialysate in modern day hemodialysis units. A possible alternative could be performing a cross-over randomized study, but in that case large wash-out periods would be required to eliminate possible carry-over effects, making the study protocol very cumbersome and increasing the chance of patient drop-out. Another alternative, in order to increase the robustness of our results, could be performing a prospective, randomized, open-label, blinded-endpoint (PROBE) trial. Moreover, the duration of intervention for each dialysate sodium level in this study was 2 weeks, which can be considered short; longer interventions are useful to further delineate the observed effects; it has to be noted however, that long-term (i.e. >1 year) studies evaluating careful interventions on ambulatory BP in hemodialysis patients are practically absent, as it is very difficult to keep all the other factors that affect BP in the average hemodialysis patient (dietary habits, dry weight, cardiovascular medications, acute illness, accelerated vascular ageing) stable for long periods in order to isolate the effects of a specific intervention. Long-term studies are needed to examine the long-term efficacy and safety of such interventions as well as possible associations with adverse outcomes in this population; the need for such studies is due to the fact that while lower sodium dialysate concentrations may be cardioprotective through lower IDWG and BP levels, it may also be associated with increased incidence of intradialytic hypotension episodes, which may have the opposite effect on cardiovascular events. Furthermore, the overall recruitment rate was rather slow (estimated ∼1 patient/5 weeks); this is inherent to the low feasibility of 72-h ABPM recordings, with almost 50% needed to repeat ABPM (20 participants) and of them, more than 50% (11 participants) had a second invalid BP recording and excluded from the study. Another explanation is that a part of the study was conducted during the first waves of the covid-19 pandemic, where the application of restrictive measures and local lockdowns limited a faster recruitment rate. Finally, the study included only patients of Caucasian origin and whether its findings apply in other populations should be further examined.

In conclusion, this pilot non-randomized study showed an increase in ambulatory BP levels during the long interdialytic interval with higher dialysate sodium concentrations in hemodialysis patients. The observed increase in IDWG with higher dialysate sodium suggests a potential volume-dependent pathogenetic mechanism. Future randomized studies are warranted to fully elucidate the effects of different dialysate sodium concentrations on ambulatory BP in patients receiving conventional hemodialysis. Furthermore, whether these short-term observations have long-term implications on hard outcomes such as all-cause and cardiovascular mortality has to be defined.

## Supplementary Material

sfae041_Supplemental_File

## Data Availability

All data generated or analyzed during this study are available through direct contact to the corresponding authors.
