# Supplementary material for: The effect of different dialysate sodium concentrations on ambulatory blood pressure in hemodialysis patients: a prospective interventional study
Source: Clin Kidney J. 2024 Feb 21;17(8):sfae041. doi: 10.1093/ckj/sfae041 (PMC11317838; doi:10.1093/ckj/sfae041)
Supplement: sfae041_Supplemental_File [file sfae041_supplemental_file.docx]

**Supplemental Material**

**Title: The effect of different dialysate sodium concentrations on ambulatory blood pressure on hemodialysis patients. A prospective interventional study**

**Authors:** Adamantia Bratsiakou^1^**,** Fotini Iatridi,^2^ Marieta Theodorakopoulou^2^, Pantelis Sarafidis^2^, Dimitrios S. Goumenos¹, Evangelos Papachristou^1^, Marios Papasotiriou^1^

**Affiliations:** 1) Department of Nephrology and Kidney Transplantation, University Hospital of Patras, Patras, Greece. 2) First Department of Nephrology, Hippokration Hospital, Aristotle University of Thessaloniki, Greece

**Supplemental Figure 1:** Study flowchart

**
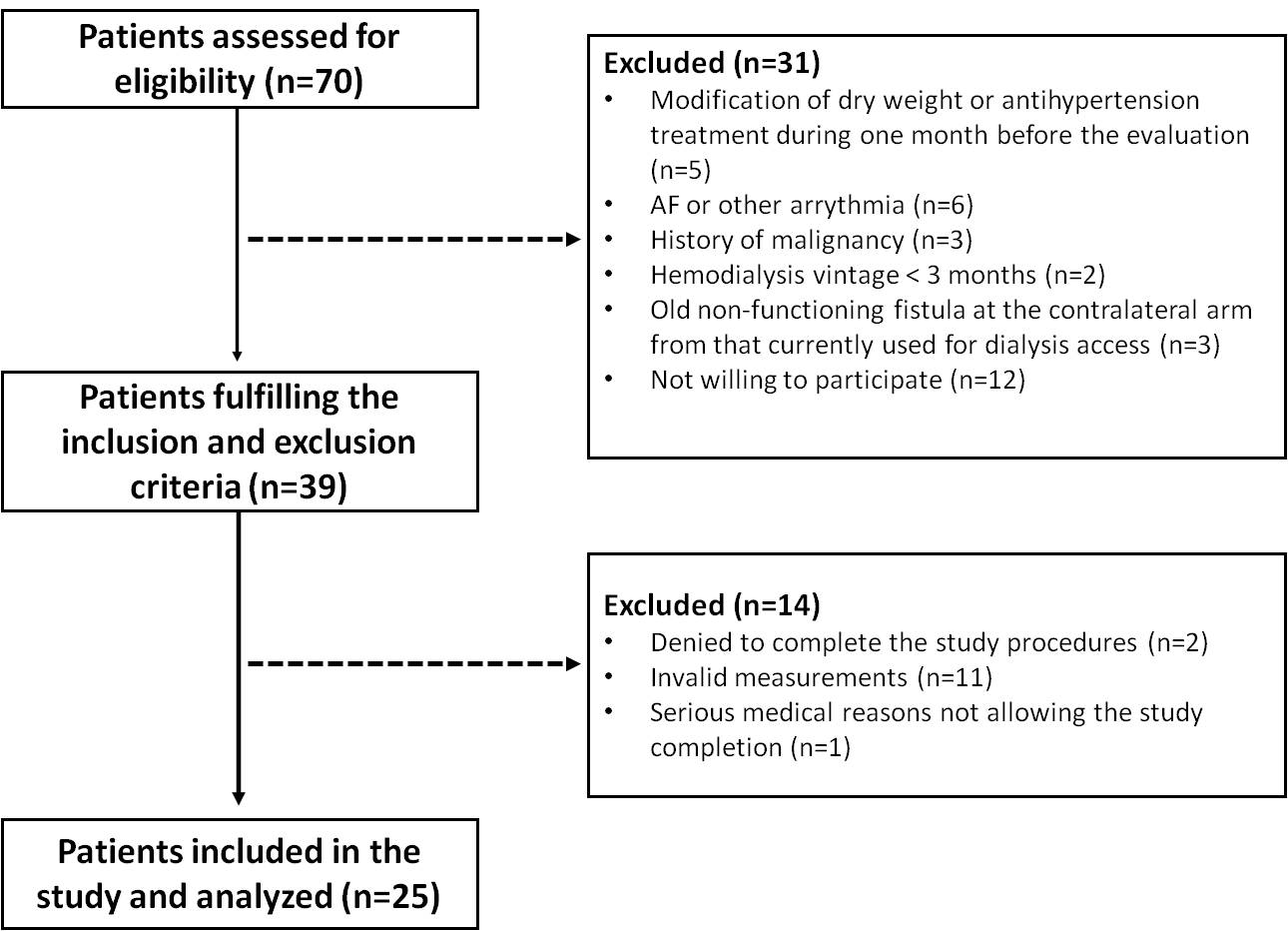
**

**Supplemental Figure 2:** Μean values of 72-h SBP and DBP and the pattern of change for each patient over the three different dialysate sodium concentrations. Data are presented as mean and standard deviation (SD)

**
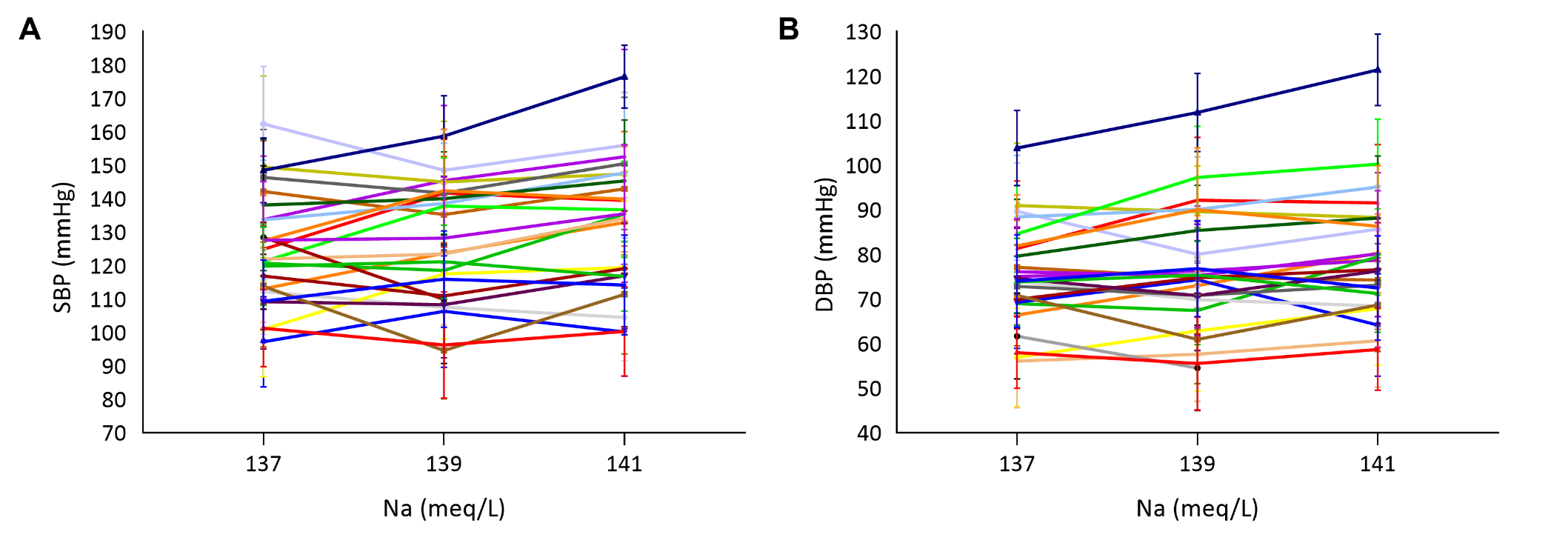
**

**Supplementary Table 1:** Differences in ambulatory BP between 1^st^ and 2^nd^ day [Δ (2-1)], 2^nd^ and 3^rd^ day [Δ (3-2)] and 1^st^ and 3^rd^ day [Δ (3-1)] with the different dialysate sodium concentrations.

| **Parameter** | **137** | **139** | **141** | **p** |
| --- | --- | --- | --- | --- |
| **Δ (2-1) 24-h SBP (mmHg)** | 2.2±6.1 | 2.5±7.7 | 4±8.1 | 0.367 |
| **Δ (2-1) 24-h DBP (mmHg)** | 0.6±4 | 0.1±4.8 | 2.2±4.2 | 0.133 |
| **Δ (2-1) day-time SBP (mmHg)** | 3.8±7.1 | 3.9±9.8 | 4.2±9.7 | 0.951 |
| **Δ (2-1) day-time DBP (mmHg)** | 1.9±4.5 | 1.4±6.4 | 2.9±5 | 0.467 |
| **Δ (2-1) night-time SBP (mmHg)** | 3.6±7.7 | 3.5±12.4 | 7.2±9.8 | 0.162 |
| **Δ (2-1) night-time DBP (mmHg)** | 3.4±5.3 | 0.2±9.1 | 4.7±7 | 0.037 |
| **Δ (3-2) 24-h SBP (mmHg)** | 6.9±6.2 | 6.4±8.1 | 5.2±5.5 | 0.611 |
| **Δ (3-2) 24-h DBP (mmHg)** | 4.5±3.8 | 3.7±5.8 | 2.3±3.9 | 0.241 |
| **Δ (3-2) day-time SBP (mmHg)** | 7.7±6.5 | 6±9.3 | 6.8±6.3 | 0.63 |
| **Δ (3-2) day-time DBP (mmHg)** | 4.9±3.9 | 3.1±6.7 | 3.2±3.6 | 0.305 |
| **Δ (3-2) night-time SBP (mmHg)** | 5.2±9.2 | 7.4±11.9 | 2.1±9.7 | 0.168 |
| **Δ (3-2) night-time DBP (mmHg)** | 3.2±5.9 | 6.2±8.7 | 0.8±7.9 | 0.031 |
| **Δ (3-1) 24-h SBP (mmHg)** | 9.1±8.9 | 8.9±11.8 | 9.4±11.1 | 0.894 |
| **Δ (3-1) 24-h DBP (mmHg)** | 5.1±4.4 | 3.9±6.8 | 4.5±5.5 | 0.6 |
| **Δ (3-1) SBP day-time (mmHg)** | 11.5±7.9 | 9.9±12.3 | 10.5±12.1 | 0.733 |
| **Δ (3-1) DBP day-time (mmHg)** | 6.9±4.6 | 4.6±7.6 | 5.9±6.2 | 0.17 |
| **Δ (3-1) SBP night-time (mmHg)** | 8.8±10.6 | 11±13.5 | 9.3±12.1 | 0.582 |
| **Δ (3-1) DBP night-time (mmHg)** | 6.7±5.7 | 6.6±9.2 | 5.5±7.7 | 0.71 |

Footnote: Data are presented as mean and standard deviation (SD)

Abbreviations: SBP, systolic blood pressure; DBP, diastolic blood pressure
